# Anterior glenoid bone reconstruction and anterior latissimus transfer for failed Latarjet associated with irreparable subscapularis tear

**DOI:** 10.1016/j.jseint.2022.08.021

**Published:** 2022-10-13

**Authors:** Jean-David Werthel, Robin Lévêque, Bassem T. Elhassan

**Affiliations:** a Department of Orthopedic Surgery, Hôpital Ambroise Paré, Boulogne-Billancourt, France; b Department of Orthopedic Surgery, Mayo Clinic, Rochester, MN, USA

**Keywords:** Failed Latarjet, Latissimus dorsi transfer, Eden-Hybinette, Anterior shoulder instability, Irreparable subscapularis

## Abstract

**Background:**

Management of combined persistent anterior glenoid bone deficiency with irreparable subscapularis tear can be very complicated and challenging especially if associated with arthritis. The objective of this study was to report the outcome of combined reconstruction of the anterior glenoid with bone autograft or allograft with additional anterior latissimus transfer to reconstruct irreparable subscapularis tear with or without humeral head replacement.

**Methods:**

Nineteen patients (average age 29 years old) who underwent open anterior glenoid bone reconstruction with iliac crest bone autograft or ostechondral bone allograft (distal tibia or glenoid allograft), with anterior latissimus transfer to reconstruct irreparable subscapularis tear with or without humeral head replacement were included in this study. Outcome measures included preoperative and postoperative pain score, visual analog scale, Subjective Shoulder Value, American Shoulder and Elbow Surgeons score, and Constant Score.

**Results:**

Out of the 19 patients, 5 patients underwent humeral resurfacing arthroplasty. Anterior glenoid bone reconstruction was performed with iliac crest bone autograft in 8 patients, glenoid osteochondral allograft in 7 patients, and tibial plafond in 4 patients. At mean 31-month follow-up of (13-63 months), 15 patients (79%) considered their shoulder stable and were able to return to their work and 14 (74%) patients returned to their sport activity. Redislocation had occurred in 1 of the 18 shoulders (5%), subluxation had occurred in 3 patients (16%) of the shoulders and apprehension was reported for 4 patients, 21% of the operated shoulders. All outcome measures showed significant improvement compared to before surgery. No intraoperative or immediate postoperative complications were observed. Four patients (21%) had to be revised to reverse shoulder arthroplasty.

**Conclusion:**

The combination of anterior latissimus transfer, anterior glenoid bone grafting with or without humeral head resurfacing is an effective salvage surgical reconstruction that can stabilize shoulders in the setting of recurrent anterior instability after a failed Latarjet with an irreparable subscapularis tear. This could be a potential alternative reconstruction option that might be offered to patients with this difficult problem. Long-term outcome is needed to better evaluate the validity of this technique.

Recurrent anterior glenohumeral instability after the Latarjet procedure is rare (1%-5%^1^^,^^17^^,^^18^^,^^25^^,^^26^). The most common etiology is persistent anterior glenoid bone loss subsequent to nonunion, osteolysis, or malposition of the coracoid bone graft.^39^ Additionally, subscapularis tear especially after open anterior shoulder stabilization can be another etiology for recurrent instability. Scheibel et al^33^ have proven that the anterior surgical approaches to the shoulder used to perform a Latarjet could lead to subscapularis dysfunction and several studies have reported high rates of subscapularis tearing after a Latarjet^23^ or an Eden-Hybinette.^34^ Persistent anterior instability has been shown to be associated with pain, limited range of motion, and progressive arthritis.^15^

On the one hand, if the reason of recurrent instability is persistent anterior glenoid bone deficiency, then, several authors^2^^,^^11^^,^^22^^,^^37^ showed good outcome with revision using iliac crest bone (ICB) block. On the other hand, if the reason of recurrent instability is a repairable subscapularis tear, then repair of the subscapularis tendon may lead to shoulder stabilization.^4^^,^^16^

However, if the subscapularis tear is deemed irreparable, then several tendon transfer options have been described to reconstruct the deficient subscapularis. These include pectoralis minor transfer ^3^^,^^30^ or pectoralis major transfer either above or below the conjoined tendon.^40^ Recent long-term studies have demonstrated excellent functional outcomes after pectoralis major transfer.^9^^,^^27^ However, it has been shown that this transfer becomes unpredictable in the setting of anterior instability or anterior subluxation.^7^ This might be due to the line of pull of the pectoralis major which comes from the anterior chest wall and therefore does not replicate the posterior line of pull of the subscapularis which lies on the posterior chest wall. In 2014, Elhassan et al ^6^ have proposed in an anatomical study to transfer a posterior muscle (the latissimus dorsi) anteriorly to the proximal-lateral aspect of the lesser tuberosity or anterior part of the supraspinatus footprint to better replicate the line of pull of the subscapularis. Since then, two clinical studies have confirmed that this option could give satisfactory clinical results ^8^^,^^28^ in patients with irreparable subscapularis tears. Management of combined persistent anterior glenoid bone deficiency with irreparable subscapularis tear can be very complicated and challenging especially if associated with arthritis. To our knowledge, the only treatment reported to this complex problem includes shoulder fusion or reverse shoulder arthroplasty (RSA).^13^ Although RSA is a reliable procedure, it may not be ideal in this young and active population and should only be reserved as a last option. Shoulder fusion has been described as a potential salvage treatment option for this complex problem. The advantage of shoulder fusion is its reliability in stabilizing the shoulder. However, shoulder fusion can be associated with high rate of complications and significant limitation of range of motion. To our knowledge, combined reconstruction of the anterior glenoid with bone autograft or allograft with additional anterior latissimus transfer to reconstruct irreparable subscapularis tear with or without humeral head replacement has not been previously reported. The objective of this study was to report the outcome of this salvage procedure. We hypothesize that this reconstruction may lead to stable shoulders with satisfactory outcome.

## Materials and methods

### Patient population

This is a retrospective study of 19 patients who underwent open anterior glenoid bone reconstruction with ICB autograft at the beginning of our experience or later with osteochondral bone allograft (distal tibia or glenoid allograft depending on availability), with anterior latissimus transfer to reconstruct irreparable subscapularis tear with or without humeral head replacement were included in this study.

All patients presented with recurrent anterior shoulder instability after failed Latarjet with associated recurrent anterior glenoid bone loss, irreparable subscapularis tear with or without arthritis. Recurrent anterior instability was defined as recurrent dislocation or subluxation after a Latarjet procedure; glenoid bone loss defined as >20% and subluxation of the humeral head were visualized on a preoperative computed tomography (CT) scan; irreparable subscapularis tear was determined by evidence of advanced fatty infiltration grade III/IV on a preoperative CT scan. This was confirmed by weakness in internal rotation with positive belly-press test, bear-hug test, and lift off test.

The average age of patients was 29 years old (range, 17-45 years), 15 males and 4 females with the dominant arm affected in 13 patients. All patients had on average 3 prior surgeries (range, 2-7 surgeries) including Latarjet procedure for anterior shoulder stabilization.

### Clinical evaluation

The authors performed full physical examination before and after surgery and at all subsequent follow-up visits. This includes examination of the range of motion, both active and passive, detailed examination of the rotator cuff with special attention to the subscapularis muscle which was assessed using the following clinical tests: belly-press test, bear-hug test, lift off test, and passive shoulder external rotation.

Collection of outcome measures included preoperative and postoperative pain score, visual analog scale (VAS), Subjective Shoulder Value (SSV), American Shoulder and Elbow Surgeons (ASES) score, and Constant Score (CS).

### Radiographic evaluation

All patients underwent plain radiographs of the shoulder (3 projections: anteroposterior, internal and external rotation, and axillary) before surgery to look for arthritic changes and at 6 weeks, 3 months, and 1 year after surgery A CT scan was performed preoperatively to determine the extent of glenoid bone deficiency, and fatty infiltration according to Goutallier and to measure humeral subluxation. Humeral subluxation was measured on reformatted axial slices using the scapulohumeral subluxation index. The scapulohumeral subluxation index uses the Friedman line as a reference, and the percentage of the humeral head posterior to the Friedman line is assessed at the longest anteroposterior diameter of the humeral head on a line perpendicular to the Friedman line. Another CT scan was obtained at 1 year postoperatively to assess positioning and healing of the iliac crest, distal tibial, or glenoid grafts.

### Surgical technique

Under general anesthesia, patients were positioned in the beach chair position. If the plan was to use the iliac crest as bone autograft, then the ipsilateral iliac crest area was included in the prepping of the operative field.

#### Approach and identification of the lesions

**Figure 1:**
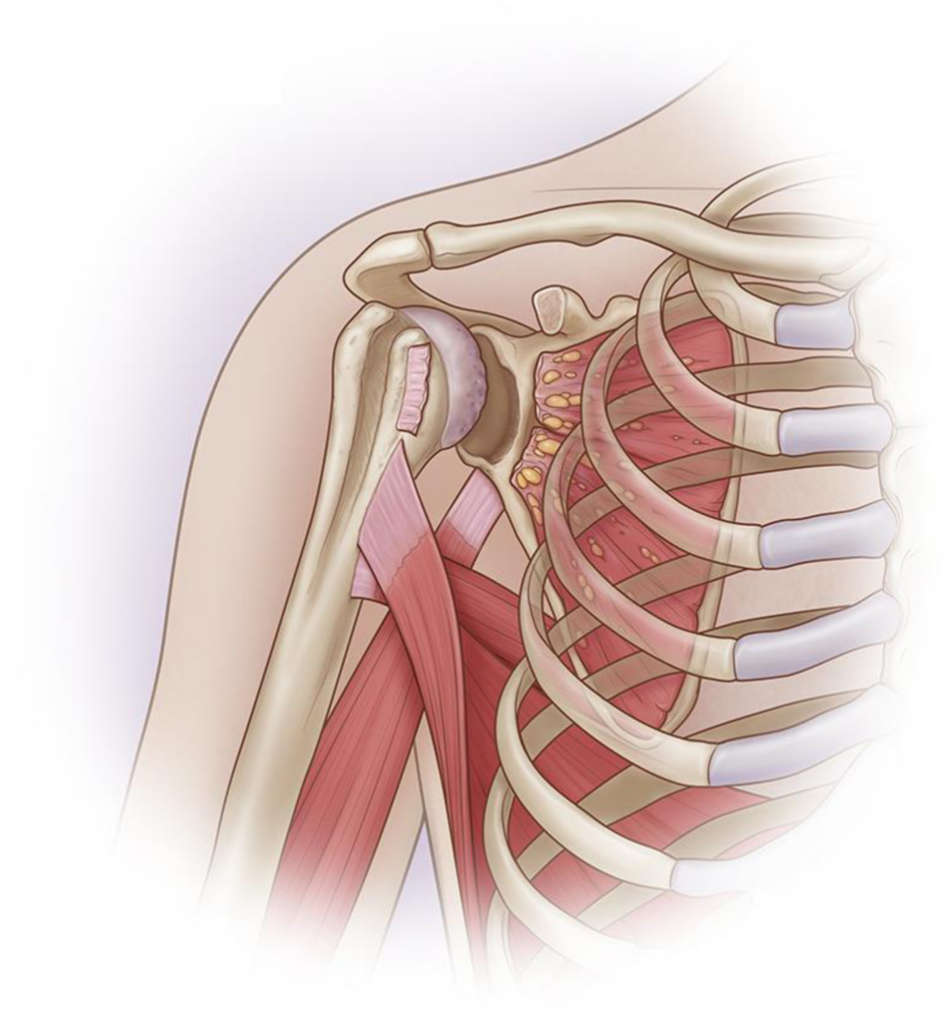
Identification of the lesions: anterior inferior bone defect of the glenoid, irreparable subscapularis tear, absence of the coracoid process.

All patients had prior scar from a prior deltopectoral approach, and this scar was reopened with slight extension distally (Fig. 1). Skin flaps were elevated and the deltopectoral interval was identified and opened. The coracoid process and conjoint tendon were no longer present in their anatomic location and could not be used as landmarks. Therefore, great care was taken during the dissection in the scar tissue anterior to the joint in order not to injure the axillary nerve.

The subscapularis and anterior capsule were no longer present and were typically replaced by thin scar tissue which was excised in order to expose the glenohumeral joint. Screws from prior Latarjet procedure were removed and the deficient anterior glenoid was exposed. The bone was débrided using a combination of osteotome and electrical burr to have a fresh even surface ready to receive the bone graft.

On the humeral side, the footprint of the subscapularis at the level of the lesser tuberosity was débrided as far proximally to the anterior footprint of the supraspinatus in preparation for later attachment of the latissimus transfer.

#### Latissimus dorsi harvesting

**Figure 2:**
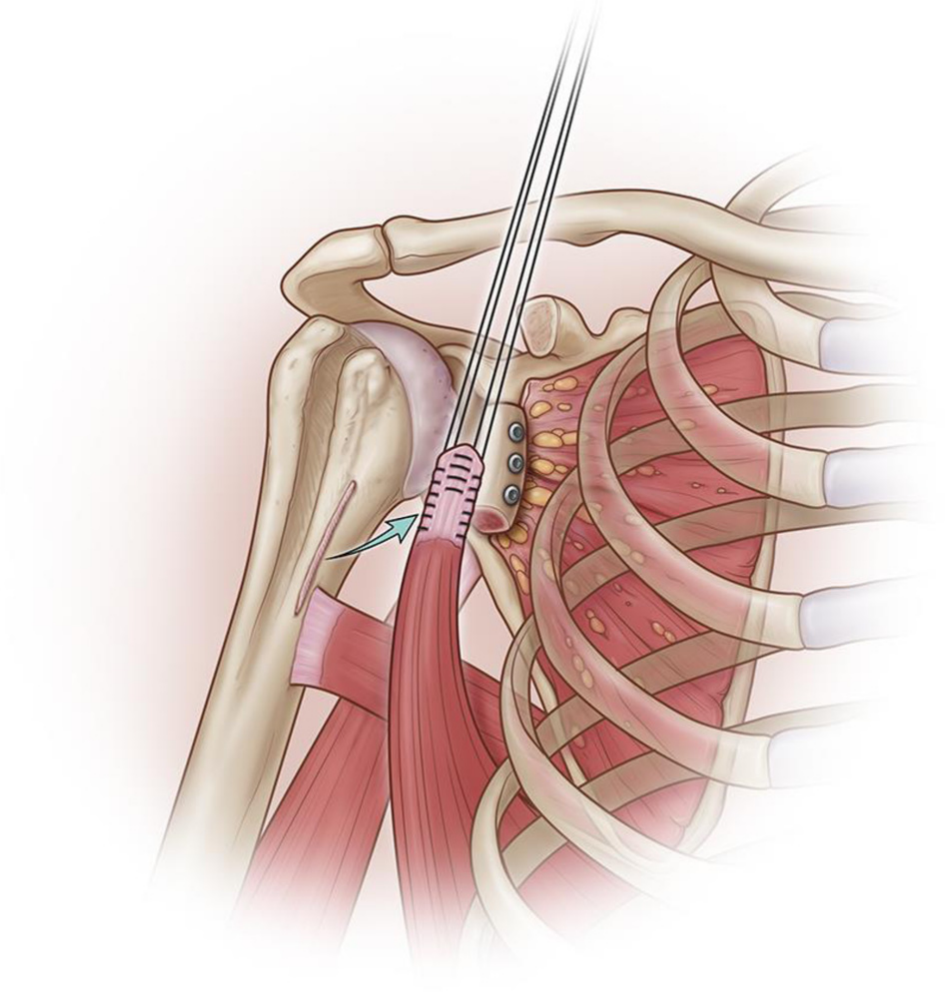
Latissimus dorsi harvesting, anterior glenoid bone reconstruction.

The pectoralis major tendon was dissected and was either retracted proximally or carefully tenotomized on its proximal 1 cm to expose the underlying latissimus dorsi tendon (Fig. 2). Retraction of the remaining pectoralis tendon while positioning the shoulder in flexion and internal rotation allowed better visualization of the distal aspect of the latissimus tendon. Since the latissimus tendon can be conjoint with the teres major tendon, we always separate these tendons proximally first because at this level, the two tendons are always clearly distinct. Separation of the two tendons was then performed from proximal to distal. Detachment of the latissimus was performed and two nonabsorbable #2 braided sutures were passed in a Krackow fashion at the superior and inferior part of the tendon on a 5-6 cm length. Traction was applied on the tendon in order to facilitate release of the latissimus dorsi from the teres major and surrounding soft tissue until sufficient excursion is obtained to allow the tendon to reach the proximal lateral aspect of the lesser tuberosity.

#### Preparation of bone graft to reconstruct the anterior glenoid

##### Iliac crest bone graft preparation (8 patients)

An incision was performed around 3-4 cm posterior to the anterior-superior iliac crest. Skin flaps were raised and the fascia over the iliac crest was identified and opened. The muscle layers were elevated to have adequate exposure of the bone. Next, using a combination of electrical saw and osteotomes a tricortical bone wedge of 25 mm in length, 15 mm in height, and 10 mm in width was harvested. The size was of course customized depending on the patient’s size and glenoid bone loss.

##### Distal tibia osteochondral allograft preparation (7 patients)

After measuring the dimensions of the anterior glenoid bone defect, the tibial plafond was cut making sure to match its concavity with the concavity of the deficient anterior glenoid.

##### Glenoid osteochondral bone allograft preparation (4 patients)

Same as for the ICB autograft and the tibial plafond osteochondral bone allograft, the size of the anterior glenoid bone defect was measured. Because of similar geometry of the glenoid allograft and native glenoid, it was easier to prepare the bone allograft, though we aimed to oversize it to make sure we had adequate amount bone for fixation.

#### Internal fixation of the anterior glenoid bone graft

**Figure 3:**
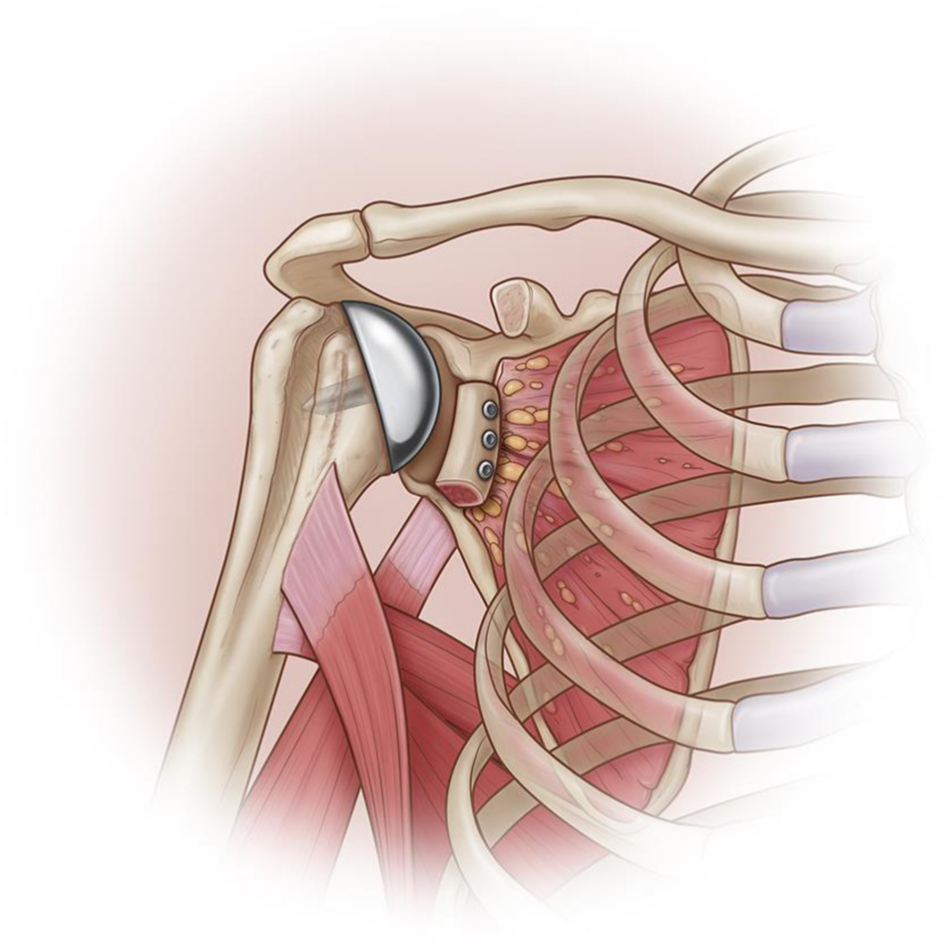
Humeral head replacement, anterior glenoid bone reconstruction.

The bone grafts were all fixed in the same manner (Figs. 2 and 3). Two or three holes (depending on the final length of the bone graft) were predrilled with a 4.5 mm drill and equally spaced in preparation for placement of 4.5 mm fully threaded cortical screws.

With adequate exposure of the glenoid, the bone graft was positioned making sure the concavity of the bone autograft (iliac crest) or the osteochondral allograft (plafond, glenoid) matched well the concavity of the native glenoid, and a 3.5 mm drill was introduced through the central hole and drilling through the glenoid was performed until the posterior cortex was penetrated. Before inserting the first screw, another drill was inserted through a different hole (either proximal or distal if we had three holes) to maintain the position and avoid malrotation of the bone. At this time, the first screw was inserted in a bicortical fashion followed by the rest of the screw(s). In all these reconstructions the objective was adequate compression of all screws and restoration of the glenoid concavity.

### Humeral head resurfacing arthroplasty

**Figure 4:**
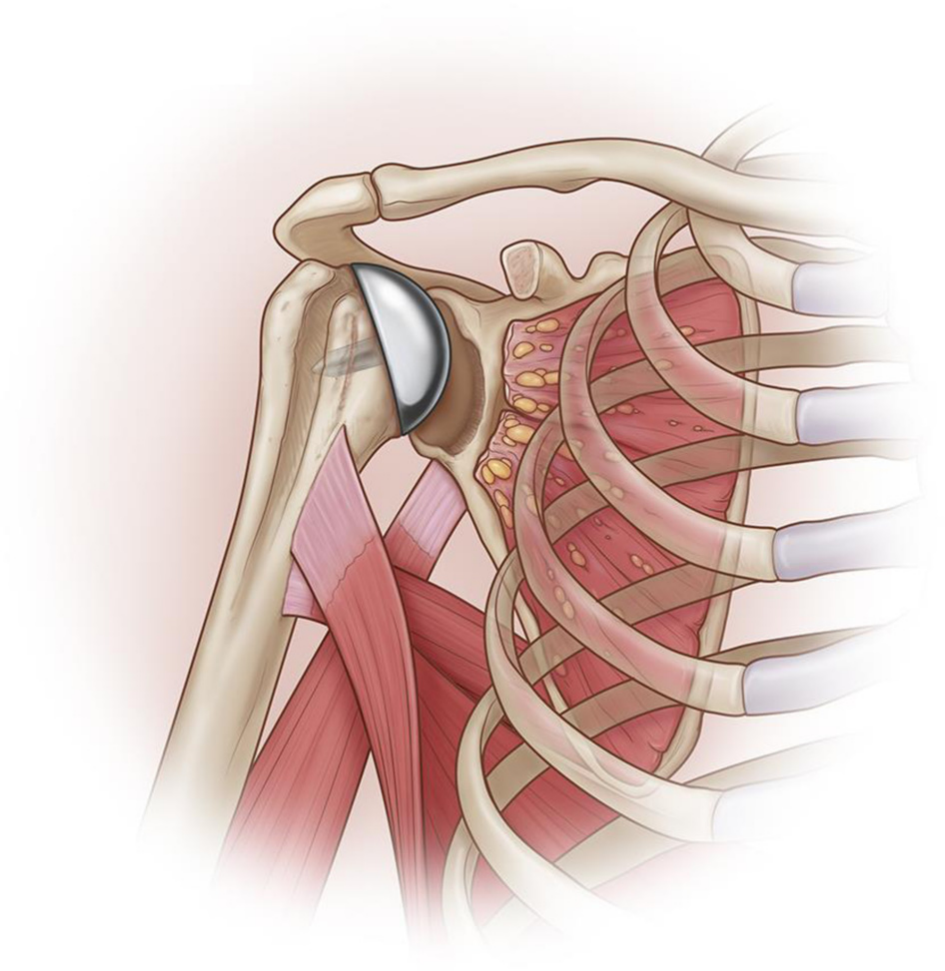
Humeral head replacement.

In 5 patients who were deemed to have advanced arthritis, (Samilson and Prieto≥2), a cementless resurfacing arthroplasty (Global CAP; DePuy, Raynham, MA, USA) was performed following the standard described technique. (Fig. 4)

### Latissimus dorsi fixation

**Figure 5:**
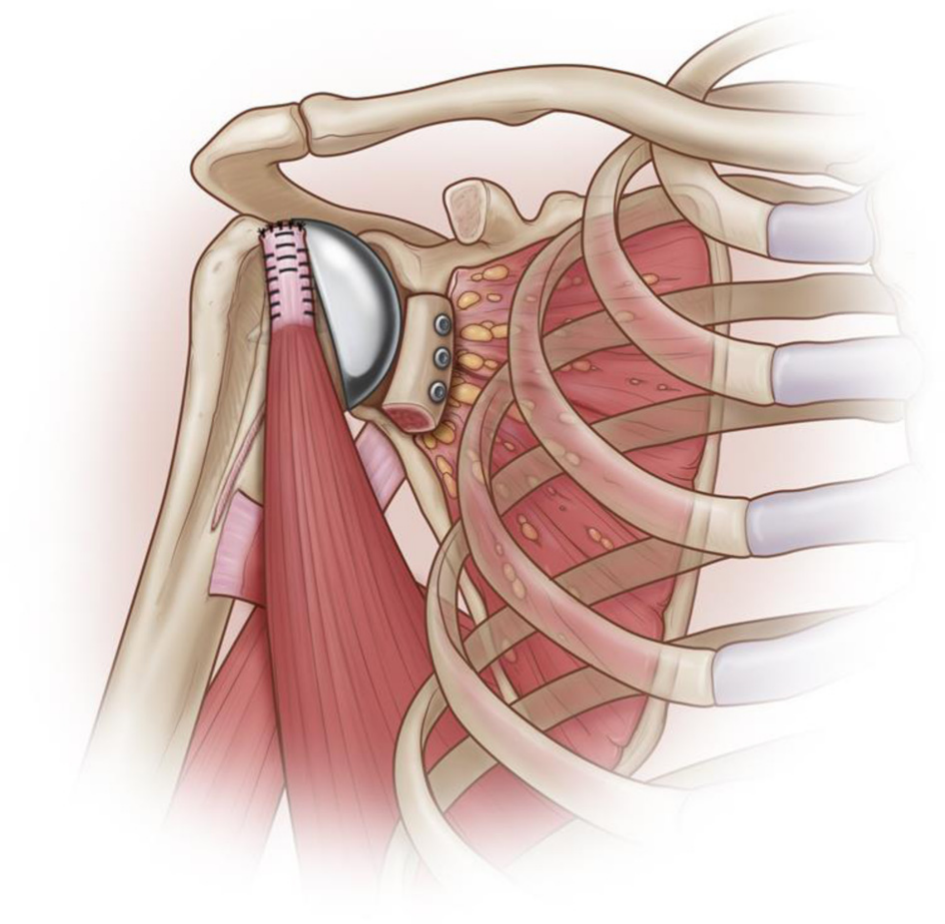
Final aspect: glenoid bone reconstruction, anterior transfer of latissimus dorsi, and humeral head replacement.

This was usually the last step to be performed (Fig. 5). If there were any remaining scarred subscapularis, we tried to repair it at this time. In most instances, there was either only some distal subscapularis remaining or nothing to be repaired. Using cortical buttons (Arthrex, Naples, FL, USA) or suture anchors (Swivelock 4.75; Arthrex, Naples, FL, USA) the two sutures placed in the latissimus are anchored to the proximal lateral aspect of the lesser tuberosity while the shoulder was kept in abduction and internal rotation.

### Postoperative management

The patient is discharged from the hospital either the same day or the day after surgery. The shoulder is immobilized in an internal rotation sling for 6-8 weeks. The sling is removed after 6 weeks for gentle passive range of motion and physical therapy is started at 8 weeks after surgery. The therapy consists of two main phases. In the first phase, active assisted range of motion is started at 8 weeks after surgery and continues for 8 weeks. During this time, we recommend avoiding stretching to decrease the chance of injuring the transferred tendon. Aqua therapy is an essential part of this phase. The second phase consists of progressive strengthening and swimming over 8 weeks. Patients are allowed full activities with no restrictions if they progressed well with no sign of failure of the shoulder reconstruction.

### Statistical analysis

Descriptive statistics were used to summarize data, with categorical data summarized as percentages and counts and continuous data as interquartile ranges. The differences between preoperative and postoperative data were compared using the 2-sample Fisher exact test for categorical variables or the Student *t* test of unequal variance for continuous and categorical variables.

## Results

Out of the 19 patients, 5 patients underwent humeral resurfacing arthroplasty. Anterior glenoid bone reconstruction was performed with ICB autograft in 8 patients, glenoid osteochondral allograft in 7 patients, and tibial plafond in 4 patients.

### Clinical evaluation

Preoperatively all patients felt their shoulder was unstable with multiple daily episodes of anterior subluxation. All patients had severe anterior apprehension and positive belly-press, bear hug, and lift off tests.

At an average follow-up of 31 months (range, 13-63 months), 15 patients (79%) considered their shoulder stable and were able to return to their work and 14 (74%) patients returned to their sport activity. Redislocation had occurred in 1 of the 18 shoulders (5%), subluxation had occurred in 3 patients (16%) of the shoulders and apprehension was reported for 4 patients, 21% of the operated shoulders.

The belly-press test was negative in 8 of 19 patients, the lift off test was negative in 12 of 19 patients, and the bear-hug test was negative in 13 of 19 patients. Passive shoulder external rotation improved from average 50^°^ (range, 40-80^°^) to average 70^°^ (range, 60-100^°^). Passive flexion improved from average 97^°^ (range, 60-100^°^) to average 135^°^ (range, 70-140^°^). Passive internal rotation improved from average L2 (range, buttock to L1) to average L4 (range, L5 to T12). Active shoulder external rotation decreased from a mean 70^°^ (range, 60-100^°^) to a mean 50^°^ (range, 40-80^°^) postoperatively. Active flexion improved from average 97^°^ (range, 60-100^°^) to average 135^°^ (range, 70-140^°^). Active internal rotation improved from average L2 (range, buttock to L1) to average L4 (range, L5 to T12).

All outcome measures showed significant improvement compared to before surgery. The mean VAS improved from average 7 (range, 5-9) to average 3 (range, 1-6) (*P* = .012). The mean SSV score improved from a mean 20% (range, 10-30) preoperatively to a mean 67% (range, 50%-80%) postoperatively (*P* = 0.02). The mean Constant Score improved from average 32 (range, 27-45) preoperatively to average 71 (range, 59-79) postoperatively (*P* = .005). The ASES score improved from average 35 (range, 27-44) preoperatively to average 74 (range, 62-82), postoperatively (*P* = .003).

### CT scan evaluation

The postoperative CT scan showed evidence of bone healing at 8 weeks postoperatively in 10 patients and 17 patients at 6 months postoperatively. Two patients had nonunion of the anterior bone graft (1 ICB autograft and 1 osteochondral glenoid bone graft).

The graft was optimally positioned (flush to the articular surface and below the equator) in all cases.

Anterior subluxation of the humeral head improved from average 17.6% (range, 5%-50%) to average 3.4% (range, 0%-20%).

Progressive arthritis was observed in 4 patients (Samilson and Prieto≥2) and 9 patients progressed to grade 1 in the classification of Samilson and Prieto at the last follow-up.

### Complications

No intraoperative or immediate postoperative complications were observed. Specifically, there was no evidence of postoperative nerve injury.

In 4 patients persistent anterior subluxation was observed which led to the progressive erosion of the anterior glenoid graft in all 4 patients, in addition, the humeral head showed signs of bone and cartilage loss in 3 patients who did not undergo resurfacing arthroplasty. These patients underwent revision to RSA after an average 8 months (range, 6-23 months) with satisfactory outcome.

## Discussion

Several studies have shown that the Eden-Hybinette was a successful solution in case of recurrent instability after a failed Latarjet.^2^^,^^22^^,^^31^ The most frequent causes of recurrence after a Latarjet are related to persistent anterior glenoid bone loss due to malpositioning, lysis, nonunion, or fracture^14^^,^^20^^,^^31^^,^^32^ of the coracoid bone graft but can also be attributed to soft tissue insufficiency mostly secondary to subscapularis tear. Indeed, anterior instability has been shown to cause lengthening and thinning of the subscapularis musculotendinous unit.^5^^,^^10^^,^^35^^,^^36^ This can lead to postoperative subscapularis disruption in around 4% of cases^12^^,^^23^ which can lead to irreparability of the subscapularis.^12^^,^^24^^,^^25^

The management of an irreparable subscapularis remains a challenge, but several tendon transfers have been proposed as treatment options. The most common of these is the pectoralis major transfer which has been described by Wirth et al^40^ in 1997 and has been reported to provide very satisfactory long-term outcomes.^9^^,^^27^ However, several authors report less reliable results using the pectoralis transfer especially if subscapularis insufficiency is associated with static anterior shoulder subluxation.^7^^,^^29^ This can probably be explained by the line of pull of this tendon transfer which is different from that of the muscle it aims to replace.^38^ Indeed, the subscapularis muscle originates from the anterior scapular body which is positioned on the posterior aspect of the chest wall whereas the sternal head of the pectoralis major originates from the anterior chest wall. Therefore, in case of existing anterior instability of the shoulder, the anterior line of action of the pectoralis major transfer could contribute to worsen the anterior instability. In order to better replicate the posterior line of pull of the subscapularis, Elhassan et al proposed in 2014,^6^ in a cadaveric study, to transfer the latissimus dorsi (posteriorly positioned on the chest) anteriorly to the proximal lateral aspect of the lesser tuberosity, next to the anterior part of the footprint of the supraspinatus. Several authors adopted this novel tendon transfer and have reported satisfactory clinical outcomes at a mean 27.8-month follow-up.^8^^,^^28^

Elhassan et al^8^ in a recent study, reported on the outcome of 56 patients who underwent latissimus dorsi transfer to reconstruct irreparable subscapularis tear. They excluded in that study patients with anterior glenoid bone defect that required bone reconstruction. Most patients had prior surgeries, average 2 (range, 1-5). Thirty patients had associated supraspinatus tear and 27 patients had anterior subluxation. At an average 13 months of follow-up, they showed that most patients had significant improvement in pain, range of motion, shoulder stability, and outcome measures. Also, they were able to reduce and stabilize the shoulder in 24 out of 27 of the patients who had anterior shoulder subluxation.

Li et al ^21^ have recently published on a patient with recurrent instability after a failed Latarjet and irreparable subscapularis successfully treated by Eden-Hybinette combined with a pectoralis major transfer. They attribute the success of the pectoralis major transfer despite anterior instability to the addition of the anterior bone graft providing greater anterior-posterior glenoid surface. However, this implies that the “center of rotation of the humeral head is shifted anteriorly” and therefore, the humeral head partially rests on the ICB graft which can be a concern for future glenohumeral arthritis.

In this study, we reported the outcome of combining anterior glenoid bone grafting with latissimus transfer, with or without humeral resurfacing arthroplasty to manage failed Latarjet with persistent anterior glenoid bone defect associated with irreparable subscapularis tear with or without associated glenohumeral joint arthritis. The presentation of this category of patients was very unique. All patients present with significant apprehension, pain, and had prior surgeries. Most of them heard from other providers before they saw us the potential options for RSA and fusion and were very pleased to hear that there was another alternative procedure that might potentially stabilize their shoulders and improve their symptoms.

Anteroposterior centering of the humeral head after our reconstruction was observed in 15 out of the 19 patients. Most of them experienced significant improvement of pain, stable range of motion and were able to go back to their work and leisure activities. In 4 patients persistent anterior subluxation was observed which led to the progressive erosion of the anterior glenoid graft in all 4 patients, in addition, the humeral showed signs of bone and cartilage loss in 3 patients who did not undergo resurfacing arthroplasty. These 4 patients underwent revision to RSA after an average 8 months (range, 6-23 months) and had satisfactory results at a mean 47-month follow-up (range, 37-66 months) with no reported complications. Mean flexion was 145° (range, 140°-150°), mean abduction was 110° (range, 100°-120°), mean external rotation was 55° (range, 50°-60°), and mean internal rotation was to the buttocks. Mean VAS score was 1.5 (range, 1-2), mean Constant Score was 79 (range, 77-80), mean ASES score was 75 (range, 74-78), and mean SSV was 83% (range, 80%-85%).

Although Lebon et al^19^ showed that revision-free survival was significantly lower in resurfacing than in hemiarthroplasty, in particular because of deleterious overstuffing of the cuff and the glenoid, we opted for resurfacing in the cases with glenohumeral joint arthritis in this very young population. The first reason was to preserve bone stock for potential subsequent revisions and to facilitate appropriate fixation of the transfer. In all cases, care was taken to downsize the humeral head in order to limit the stress on the native glenoid and on the remaining rotator cuff tendons. Tension on the subscapularis repair was not a concern in this population as it was already torn and irreparable.

Limitations of the present study include its retrospective design, the small number of patients and the large number of different variables (three different types of bone grafts and resurfacing or not). In addition, the absence of a matched cohort of patients do not allow to determine whether a latissimus dorsi transfer alone or an Eden-Hybinette alone would be sufficient to stabilize the shoulder of this challenging group of patients. However, patients presenting with recurrent instability after a failed Latarjet combined with an irreparable subscapularis are rare and no gold standard treatment exists for these patients to our knowledge.

## Conclusion

The combination of anterior latissimus transfer, anterior glenoid bone grafting with or without humeral head resurfacing is an effective salvage surgical reconstruction that can stabilize shoulders in the setting of recurrent anterior instability after a failed Latarjet with an irreparable subscapularis tear. This could be a potential alternative reconstruction option that might be offered to patients with this difficult problem. Long-term outcome is needed to better evaluate the validity of this technique.

## Disclaimers

Funding: No funding was disclosed by the authors.

Conflicts of interest: The authors, their immediate families, and any research foundation with which they are affiliated have not received any financial payments or other benefits from any commercial entity related to the subject of this article.
